# The genus *Alphitobius* Stephens (Coleoptera, Tenebrionidae, Alphitobiini) in Africa and adjacent islands

**DOI:** 10.3897/zookeys.415.6676

**Published:** 2014-06-12

**Authors:** Wolfgang Schawaller, Roland Grimm

**Affiliations:** 1 Staatliches Museum für Naturkunde, Rosenstein 1, D-70191 Stuttgart, Germany; 2 Unterer Sägerweg 74, D-75305 Neuenbürg, Germany

**Keywords:** Tenebrionidae, Alphitobiini, *Alphitobius*, taxonomy, new species, new synonym, new combination, Africa, species key

## Abstract

All species of the genus *Alphitobius* Stephens, 1829 (Alphitobiini Reitter, 1917, subfamily Tenebrioninae Latreille, 1802) from Africa and adjacent islands are revised. New species: *Alphitobius capitaneus* **sp. n.** from Kenya. New synonyms: *Cryptops ulomoides* Solier, 1851, **syn. n.** of *Alphitobius diaperinus* (Panzer, 1796); *Alphitobius rufus* Ardoin, 1976, **syn. n.** of *Alphitobius hobohmi* Koch, 1953); *Peltoides (Micropeltoides) crypticoides* Pic, 1916, **syn. n.** of *Peltoides (Micropeltoides) opacus* (Gerstaecker, 1871), **comb. n.** Homonym: *Alphitobius ulomoides* Koch, 1953 = *Alphitobius arnoldi* **nom. n.** New combinations from *Alphitobius*: *Ulomoides basilewskyi* (Ardoin, 1969), **comb. n.**; *Peltoides (Micropeltoides) opacus* (Gerstaecker, 1871), **comb. n.** Figures of all examined species are added and a species key is compiled.

## Introduction

Two species of the genus *Alphitobius* Stephens, 1829 (Alphitobiini Reitter, 1917, subfamily Tenebrioninae Latreille, 1802), namely *Alphitobius diaperinus* (Panzer, 1796) and *Alphitobius laevigatus* (Fabricius, 1781), have a cosmopolitan synanthropic distribution. All the other species were described from mature habitats in Africa south of the Sahara, so probably this is also the native area of both synanthropic species. Gebien (1921) presented the first key of the African species, including species of the genus *Diaclina* Jacquelin du Val, 1861. Additional species were added by Koch (1953), Ardoin (1958, 1963a, 1969, 1976), Ferrer (1983), and Bremer (1985). The goal of the present paper is a taxonomic revision of the African species, including the description of a new species, the recognition of new synonymies, a new name for a homonym, the transfer of two species from *Alphitobius* to *Ulomoides* and *Peltoides* respectively, providing also figures of all examined species, and compiling of a species key as well. Unfortunately, two taxa (*Alphitobius grandis* Fairmaire, 1897 and *Alphitobius limbalis* Fairmaire, 1901) from Madagascar remained unknown to the authors.

The separation of *Alphitobius* from *Diaclina* was doubtful for a long time. Gebien (1921) separated both by the width of the genal canthus (wider than eyes in *Alphitobius*, narrower or as wide as eyes in *Diaclina*). However, some taxa described under *Alphitobius* have the canthus not broader than eyes (for example *Alphitobius lamottei* Ardoin, 1963, see also in species key of Bremer and Girard 1996). Only recently, Matthews and Bouchard (2008) defined the Alphitobiini, separated this tribe from the Diaperini, and discussed also a few differences between *Alphitobius* and *Diaclina*.

Some additional taxa were originally described under *Alphitobius*, but were assigned in the meantime to other genera, and are therefore not included herein. *Ulomoides cinctellus* (Fairmaire, 1902) (Madagascar), *Diaclina parallela* (Thomson, 1858) (Guinea), *Micropedinus pullulus* (Boheman, 1858) (Hongkong), *Menimus nitidulus* (Motschulsky, 1859) (Sri Lanka), *Menimus punctulatus* (Motschulsky, 1859) (Sri Lanka), *Ulomoides suffusus* (Wollaston, 1867) (Cape Verde), *Uloma sulcipennis* (Thomson, 1858) (Gabon), and *Ulomoides xamiaphilus* (Carter, 1920) (Australia). *Alphitobius distinguendus* Fairmaire, 1869 turned out to be a synonym of *Cenoscelis pulla* (Erichson, 1843). Herein, we transfer one additional species from *Alphitobius* to *Ulomoides*: *Ulomoides basilewskyi* (Ardoin, 1969), comb. n., and one from *Alphitobius* to *Peltoides (Micropeltoides)*: *Peltoides opacus* (Gerstaecker, 1871), comb. n.

### Depositories

CNC Canadian National Collection of Insects, Ottawa, Canada

CRA Collection Dr. Rolf Aalbu, Dorado Hills, USA/California

CRG Collection Dr. Roland Grimm, Neuenbürg, Germany

MNB Museum für Naturkunde, Berlin, Germany

MNHN Muséum National d’Histoire Naturelle, Paris, France

MRAC Museé Royal de l’Afrique Centrale, Tervuren, Belgium

NHMB Naturhistorisches Museum, Basel, Switzerland

NMP National Museum, Department Entomology, Prague, Czech Republic

SMNS Staatliches Museum für Naturkunde, Stuttgart, Germany

TMSA Ditsong National Museum of Natural History, Pretoria, South Africa

ZSM Zoologische Staatssammlung, Munich, Germany

## The African species of *Alphitobius*

### Alphitobius acutangulus

Gebien, 1921

http://species-id.net/wiki/Alphitobius_acutangulus

Figs 12 , 18

#### Type specimens examined.

Senegal, no further data, holotype NHMB (sex not examined).

#### New material.

Sudan, Dilling, 20.–22.III.1914, leg. Ebner, 1 ex. NHMB. – Sudan, N Darfur Prov., El Geneina, 4.–18.VI.1979, leg. I. Abuzinid, 8 ex. TMSA, 1 ex. CRG, 1 ex. MNB, 1 ex. SMNS (det. Bremer). – Burkina Faso (labelled as Ob. Volta), Pundu, Olsufiew, no further data, 4 ex. TMSA. – Chad, Massaguet, without date, leg. H. Franz, 1 ex. NHMB. – Chad, Deressia, near Lai, without date, leg. H. Franz, 1 ex. NHMB.

#### Type locality.

“Senegal”.

#### Distribution.

Senegal (Gebien 1921, Koch 1953); Sudan (Bremer and Girard 1996); Burkina Faso, Chad (new records).

### Alphitobius arnoldi nom. n.

Figs 4 , 19

Alphitobius ulomoides Koch, 1953 (homonym, not *Cryptops ulomoides* Solier, 1851, syn. n.)

#### Type specimens examined.

Zimbabwe (labelled as S Rhodesia), Bulawayo, leg. G. Arnold, holotype TMSA (sex not examined).

#### New material.

Somalia, Car-Car Mts., IX.1959, leg. C. Koch, 2 ex. TMSA (det. Ferrer). – Somalia, Gardo, 810 m, 22.X.1957, leg. G. Scortecci, 1 ex. TMSA. – Kenya, Witu, Lamu, Wangi, without date, leg. G. Denhardt, 1 ex. ZSM, 3 ex. MNB (det. Bremer). – Tanzania, Mgorogoro Prov. 10 km N Mikumi, 11.I.2007, leg. F. Kantner, 1 ex. SMNS.

#### Type locality.

“Bulawayo”.

#### Remarks.

*Cryptops ulomoides* Solier, 1851 from Chile is a junior synonym of *Alphitobius diaperinus* (Panzer, 1796) (see below). Thus *Alphitobius ulomoides* Koch, 1953 is a homonym and must have a new name, *arnoldi* nom. n.

#### Etymology.

The new name is derived in honor of George Arnold (1881–1963), former curator in the “Rhodesia Museum” (now Natural History Museum of Zimbabwe, Bulawayo), specialist of African Hymenoptera, and collector of the holotype.

#### Distribution.

Zimbabwe, Congo (Koch 1953); Somalia, Kenya, Tanzania (new records).

**Figures 1–4.**
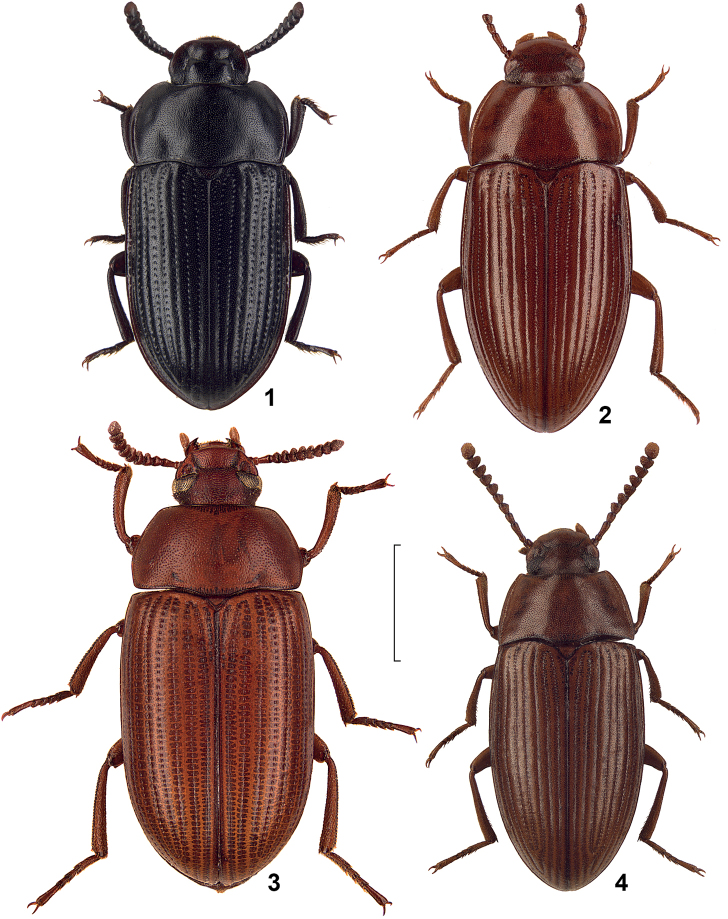
Dorsal view of African species of the genus *Alphitobius* and *Ulomoides*. **1** *Alphitobius crenatus*, non-type Madagascar, SMNS **2** *Alphitobius lamottei*, non-type Central African Republic, SMNS **3** *Ulomoides basilewskyi* comb. n., holotype, MRAC **4** *Alphitobius arnoldi* nom. n., non-type Tanzania, SMNS. Scale: 2 mm.

### Alphitobius capitaneus sp. n.

http://zoobank.org/C18D8875-7C0A-46B4-8195-313B8DE1A2BE

http://species-id.net/wiki/Alphitobius_capitaneus

Figs 16 , 20

#### Type specimens.

Holotype male: Kenya, Samburu Nature Reserve, Samburu River Lodge, 28.III.1988, leg. R. Grimm, CRG.

#### Diagnosis.

*Alphitobius capitaneus* sp. n. is distinguished from its congeners by size and shape of body. A similar body shape, especially the shape of pronotum has *Alphitobius lamottei* Ardoin, 1963, but this species is smaller (body length ≤ 7.0 mm) and differs by the reddish colour, by much finer punctation of dorsal surface, by longer and less distinct serrate antennae with basal antennomeres more elongated, and by the somewhat more stretched and apically narrowed apicale of aedeagus.

#### Description.

Body length 8.8 mm, width at widest point behind middle of elytra 3.6 mm. Elongate, blackish brown, matt; borders of pronotum, lateral borders of elytra, sutural interval, and scutellum paler reddish brown. Complete dorsal surface with very fine and dense punctation, punctures bearing a minute seta. Head sub-trapezoidal; outline continuous, not interrupted between clypeus and frons; apical margin of epistome shallowly emarginate in the middle; fronto-clypeal suture complete and linearly impressed. Eyes large, constricted by genal canthus, dorsal part smaller than ventral part. Genal canthus hardly projecting beyond contours of eyes, with the outlines nearly continuous with the outlines of the latter. Tempora strongly narrowed towards neck. Antennae thickened, not reaching the base of pronotum, with the seven distal antennomeres forming a kind of club; 2^nd^ antennomere wider than long; 3^rd^ elongate, one and a half times as long as wide; 4^th^ only slightly, 5^th^ to 10^th^ distinctly wider than long and distinctly serrate; distal antennomere rounded, as wide as long. Pronotum transverse, width/length ratio 1.7; transverse convex, widest at base, shallowly arcuate narrowing to apex. Anterior margin shallowly emarginate, basal margin bisinuate, all margins finely bordered; lateral margins separated from discal convexity by a narrow submarginal depression. Anterior and posterior corners rectangular. Propleura densely covered with small seta bearing tubercles, only along outer margins nearly smooth. Prosternum rugosely punctured, prosternal apophysis bent down behind procoxae. Elytra convex, elongate oval with subparallel sides and densely punctured striae; scutellar striole absent; intervals much broader than striae, nearly flat on disc, becoming more and more convex laterally and distally; lateral margins in dorsal view visible nearly over entire length, only concealed around apex; base as wide as base of pronotum; humeral angles obtuse, distinct. Scutellum large, triangular. Mesoventrite roughly punctured, with shiny median carina in basal part; triangular apophysis raised upwards and excavate. Metaventrite shiny, with fine median sulcus, finely punctured on disc, somewhat more coarsely punctured laterally. Abdominal ventrites with very dense and fine punctation throughout, basally and laterally longitudinally wrinkled. Tibiae gradually and faintly dilated towards apex, without modifications. Aedeagus as in Fig. 20.

#### Etymology.

Capitaneus (Latin) means conspicuous by greatness, refers in this case to the body size.

### Alphitobius crenatus

(Klug, 1834)

http://species-id.net/wiki/Alphitobius_crenatus

Figs 1 , 21

Phaleria crenata Klug, 1834
Cataphronetis luctuosa Fairmaire, 1869, syn.

#### New material.

Madagascar, no further data, 2 ex. TMSA. – Madagascar, Ambaton, no further data, 1 ex. SMNS. – S Madagascar, Midongy, no further data, 3 ex. SMNS. – C Madagascar, Katsepy (Majunga), 24.–31.XII.1997, leg. P. Pacholátko, 1 ex. SMNS. – Madagascar, Antananarivo Distr., Moramanga, 12.II.1995, leg. I. Jeniš, 1 ex. ZSM. – Madagascar, Toliaro Prov., Andohahela NP, Forêt d’Ambohibory, 300 m, 16.–20.I.2002, leg. Fisher, Grifswold et al., 1 ex. CRA. – Madagascar, Toliaro Prov., Cap Sainte Marie, 200 m, 11.–15.II.2002, leg. Fisher, Grifswold et al., 3 ex. CRA. – Madagascar, Toliara Prov., Forêt de Tsinjoriaky, 70 m, 6.–10.III.2002, leg. Fisher, Grifswold et al., 1 ex. CRA. – Madagascar, Toliara Prov., Tsimanampetsotsa NP, 25 m, 18.–22.III.2002, leg. Fisher, Grifswold et al., 1 ex. CRA.

#### Type locality.

“Madagascar” (*crenata*), “Nossi-Bé” (*luctuosa*).

#### Distribution.

Madagascar (type locality), eastern Africa, Comores, Seychelles, Aldabra Islands (Koch 1953).

### Alphitobius diaperinus

(Panzer, 1796)

http://species-id.net/wiki/Alphitobius_diaperinus

Figs 15 , 22

Tenebrio diaperinus Panzer, 1796
Tenebrio ovatus Herbst, 1799, syn.
Uloma opatroides Brullé, 1838, syn.
Cryptops ulomoides Solier, 1851, syn. n. (not homonym *Alphitobius ulomoides* Koch, 1953, *arnoldi* nom. n.)
Crypticus longipennis Walker, 1858, syn.
Phaleria rufipes Walker, 1858, syn.
Proselytus caffer Fåhraeus, 1870, syn.

#### Type specimens examined.

Chile (labelled as Chili), Valparaiso, Gay 15-43, 1 syntype of *Cryptops ulomoides* Solier, 1851, MNHN, designated herewith as lectotype.

#### New material.

Somalia, Genale, 1935, leg. R. Ciferri, 3 ex. TMSA. – Sudan, Mt. Sangha, no further data, leg. Škulina, 1 ex. NMP. – Sudan, Wad Medani, 21.XII.1952, leg. W. Büttiker, 1 ex. TMSA. – Ghana, Ashanti Region, Kumasi, Nhiasu, 16.V.1987, leg. S. Endrödy-Younga, 1 ex. TMSA. – Nigeria, Ile-Ife, 7.VII.1988, leg. F.-T. Krell, 10 ex. SMNS. – Liberia, Bong Town, 23.III.1988, leg. F.-T. Krell, 3 ex. SMNS. – Ivory Coast, Adiopodoumé, 11.V.1988, leg. F.-T. Krell, 8 ex. SMNS. – Cameroon, Buea, 11.II.1980, leg. H. Schmalfuss & M. Schlegel, 6 ex. SMNS. – Guinea, Kouroussa, 16.XI.1995, leg. U. Lange, 1 ex. SMNS. – Congo, Tshuapa, Flandria, 1946/1947, leg. P. Hulstaert, 3 ex. TMSA. – Uganda, Kampala, V.1927, leg. H. Hargreaves, 1 ex. TMSA (det. Bryant). – Kenya, no further data, leg. Škulina, 1 ex. NMP. – Kenya, Voi (Tsavo), 8.–10.XI.1996, leg. M. Snížek, 1 ex. CRG. – Zambia, 30 km NE Livingstone, 1.XI.2002, leg. F. Wachtel,1 ex. CRG. – Zambia, 30 km NW Sesheke, 13.I.2010, leg. P. Schüle, 1 ex. SMNS. – Zambia, Western Prov., 7.6 km S Ushaa, 1050 m, 2.XII.2010, leg. F. Génier, 1 ex. CNC. – Angola, Cunene Prov., 10 km N Humbe, 3.XI.2011, leg. P. Schüle, 1 ex. SMNS. – Angola, Oshikoto Prov., 9 km S Ondangwa Nakambale Museum, 25.XI.2012, leg. P. Schüle, 1 ex. SMNS. – Namibia, Abachaus, XII.1949, leg. G. Hobohm, 1 ex. TMSA. – Namibia, Kaokoveld, 13 km W Ehombe Mt., 11.II.1975, leg. S. Endrödy-Younga & Schulze, 1 ex. TMSA. – Namibia, Etosha NP, Halali, 16.–17.XII.1993, leg. M. Uhlig, 1 ex. MNB. – NW Namibia, Epupa Falls, 660 m, 11.–12,.IV.2005, leg. W. Schawaller, 1 ex. SMNS. – S Namibia, Naukluft Park East, 1500 m, 7.–10.II.2010, leg. W. Schawaller, 1 ex. SMNS. – Botswana, Okavango, Maxwee Lagoon, VIII.1976, leg. Russel-Smith, 1 ex. TMSA. – South Africa, Northern Cape, Pofadder, 760 m, 4.X.1990, leg. W. Wittmer, 1 ex. NHMB. – South Africa, Limpopo, Krüger NP, 18.VI.1990, leg. L. Braack, 3 ex. TMSA. – South Africa, Limpopo, Amatola Farm NE Vivo, 1000 m, 15.–17.XII.2003, leg. W. Schawaller, 4 ex. SMNS. – South Africa, Gauteng, Pretoria Distr, Roodeplat, 8.–10.X.1960, leg. Neubecker, 1 ex. TMSA. – South Africa, Gauteng, Ezemvelo NR, 26.I.2004, leg. TMSA staff, 3 ex. TMSA. – South Africa, KwaZulu-Natal, Hluhluwe, 29.XI.1992, leg. S. Endrödy-Younga, 1 ex. TMSA. – South Africa, KwaZuluNatal, Ndumo, 21.XI.2002, leg. J. Harrison & R. Müller, 1 ex. TMSA. – South Africa, KwaZulu-Natal, Kosi Bay NR, 11.–17.XI.2002, leg. W. Schawaller, 1 ex. SMNS. – South Africa, Free State, Farm Abel 52, 4 km E Parys, 12.–13.I.1992, leg. M. Krüger, 6 ex. TMSA. – South Africa, Northern Cape, 70 km S Olifantshoek, Witsand NR, 1200 m, 4.–7.II.2012, leg. W. Schawaller, 2 ex. SMNS. – Madagascar, Kirindy Forest, 21.XI.1998, leg. R. Müller, 2 ex. TMSA. – Madagascar, Tsaratanana, Antsirasira, Morwato West, 26.XI.–3.XII.2001, leg. V. Dolin, 1 ex. SMNS. – Rodrigues Island, Anse aux Anglais, Port Mathurin, 22.IX.1995, leg. R. Fricke, 4 ex. SMNS.

#### Type locality.

“Germanica” (*diaperinus*); “Valparaiso” (*ulomoides*).

#### Synonymy.

Examination of the syntype of *Cryptops ulomoides* Solier, 1851, shows a complete correspondence with *Alphitobius diaperinus*. The genus *Cryptops* was considered as synonym of *Alphitobius* since a long time (for example in the world catalogue of Gebien 1940), but the species *ulomoides* Solier, 1851 was not formally synonymised with *diaperinus* Panzer, 1796, so far. In consequence, *Alphitobius ulomoides* Koch, 1953 is a homonym and needs a new name, *arnoldi* nom. n. (see above).

#### Distribution.

Cosmopolitan.

### Alphitobius grandis

Fairmaire, 1897

http://species-id.net/wiki/Alphitobius_grandis

#### Remarks.

Material of this taxon is unknown to the authors. The type is said to be 9 mm long (Fairmaire 1897), the antennae are relatively short (“assez courtes et robustes”), the anterior corners of the pronotum are rectangular (“presque droit”), and the elytra have weak striae with large punctures (“stries assez peu profondes, mais fortement ponctués”).

#### Type locality.

“Madagascar”.

#### Distribution.

Madagascar.

### Alphitobius hobohmi

Koch, 1953

http://species-id.net/wiki/Alphitobius_hobohmi

Figs 5 , 23

Alphitobius rufus Ardoin, 1976, syn. n.

#### Type specimens examined.

Namibia (labelled as SWA), Abachaus, XII.1946, leg. G. Hobohm, holotype, 1 paratype *hobohmi* TMSA (sex not examined). – Tanzania, Mts. Uluguru, Morogoro Campus Fac. Agriculture, 600 m, V./VI.1971, leg. J. Debecker, ♀ holotype *rufus* MRAC.

#### New material.

Ethiopia, Hararge Prov., Bisidimo, 1500 m, V.–VII.1984, leg. V. Meyer, 2 ex. ZSM. – Kenya, Meru Distr., Materi (Mitunguu), 800 m, 8.IV.1987, leg. R. Mourglia, 1 ex. SMNS (*rufus* det. Bremer). – Kenya, Meru Distr., Mojwa, 1300 m, 3.IV.1987, leg. R. Mourglia, 1 ex. ZSM (*rufus* det. Bremer). – Tanzania, Moschi, no further data, 1 ex. ZSM. – Tanzania, Manyara Lake, XII.1961, leg. H. & B. Frey, 1 ex. NHMB. – Namibia, Windhuk, 1906, leg. F. Kunze, 1 ex. MNB (det. Ferrer). – Angola, Blé Prov., Chissamba Mission Station, 1440 m, 9.XI.2011, leg. R. Müller, 1 ex. TMSA. – Angola, Huila Prov., 10 km SW Cacula, 1550 m, 4.–6.XI.2011, leg. R. Müller & P. Schüle, 1 ex. TMSA, 3 ex. SMNS. – Angola, Huila Prov., 15 km S Caluquembe, 1620 m, 6.XI.2011, leg. R. Müller, 1 ex. TMSA. – Angola, Huila Prov., 3.5 km SW Negola, 8.XII.2012, leg. P. Schüle, 2 ex. SMNS, 2 ex. CRG. – South Africa, Limpopo, Naboomspruit, Torino Ranche, 15.I.1990, leg. S. Endrödy-Younga, 1 ex. TMSA. – South Africa, Limpopo, 10 km N Modimolle (Nylstroom), 1300 m, 10.XII.2008, leg. R. Müller, 1 ex. TMSA. – South Africa, Free State, Bothaville, Vaal River, 16.I.2003, leg. M. Snižek, 1 ex. SMNS. – South Africa, KwaZulu-Natal, SW Magudu, 4.–5.I.2009, leg. R. Müller & P. Schüle, 2 ex. SMNS, 2 ex. TMSA. – South Africa, KwaZulu-Natal, Ndumo NR, 100 m, 10.XII.2010, leg. R. Müller, 1 ex. TMSA.

#### Type localities.

“Abachaus, Otjiwarongo” (*hobohmi*), “Morogoro” (*rufus*).

#### Synonymy.

The Type specimens examined of *Alphitobius hobohmi* and *Alphitobius rufus*, as well as several non-type specimens from Namibia and adjacent Angola (near type locality of *hobohmi*), and from Tanzania and Kenya (near type locality of *rufus*) show no distinct external differences. The aedeagi of type specimens can not be compared, because the holotype of *rufus* is a female. Nevertheless, *Alphitobius rufus* is considered as a junior synonym of *Alphitobius hobohmi*.

#### Remarks.

In some localities (for example Modimolle and Magudu) *Alphitobius hobohmi* was collected together with *Alphitobius viator*.

#### Distribution.

Namibia (Koch 1953, Ferrer 2004); Tanzania, Kenya (Ardoin 1976); Ethiopia, Angola, South Africa (new records).

**Figures 5–8.**
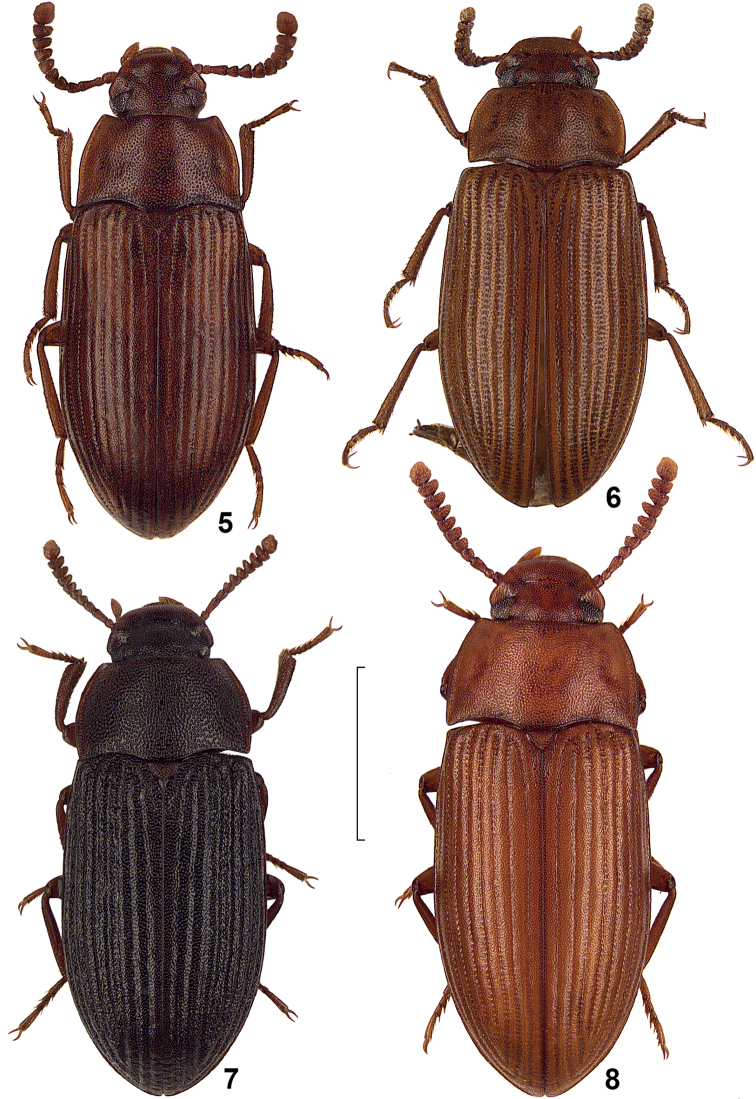
Dorsal view of African species of the genus *Alphitobius*. **5** *Alphitobius hobohmi*, non-type Angola, SMNS **6** *Alphitobius leleupi*, paratype, TMSA **7** *Alphitobius rugosulus*, non-type Tanzania, SMNS **8** *Alphitobius viator*, non-type RSA, SMNS. Scale: 2 mm.

### Alphitobius karrooensis

Koch, 1953

http://species-id.net/wiki/Alphitobius_karrooensis

Figs 13 , 24

#### Type specimens examined.

Eastern Cape, Willowmore, 20.III.1919, leg. H. Brauns, holotype, 3 paratypes TMSA.

#### New material.

Tanzania, Tabora, leg. Holtz, 1 ex. MNB (det. Bremer as *Alphitobius leleupi*). – Zambia, Western Prov., 3.8 km E Namushakende, 1100 m, 29.XI.2010, leg. F. Génier, 1 ex. CNC. – Zambia, Western Prov., 7 km S Mukokwa, 1100 m, 1.XII.2010, leg. F. Génier, 1 ex. CNC, 1 ex. SMNS. – Botswana, Okavango, Thamalakane, XII.1973, leg. P. Reavel, 1 ex. CRG. – Botswana, Chizwina, Francistown-Mosetse, 5.V.1995, leg. J. Harrison, 1 ex. SMNS. – Namibia (labelled as S. W. Africa), 3 miles NE Waterberg, 1420 m, 21.XII.1966, leg. E. S. Ross & K. Lorenzen, 1 ex. CRA. – Namibia (labelled as S. W. Africa), Okahandja, Farm Okaundua, 21.–29.XI.1933, leg. W. Krieg, 1 ex. NHMB. – South Africa, Northwest Prov., Bloemhof, 24.IV.1961, leg. F. Zumpt, 2 ex. NHMB. – South Africa, Kalahari Gemsbok Park, Nossob River, V.1956, TMSA Expedition, 12 ex. TMSA, 2 ex. SMNS. – South Africa, Kalahari Gemsbock Park, Mata-Mata, 18.XII.1974, leg. S. Endrödy-Younga, 1 ex. TMSA. – South Africa, Limpopo, Waterberg, Geelhoutbush Farm, 3.X.1995, leg. S. Endrödy-Younga & C. Bellamy, 2 ex. TMSA. – South Africa, Northern Cape, Garies, 14.XI.1948, leg. C. Koch, 1 ex. TMSA. – South Africa, Northern Cape, Witsand NR, 1160 m, 5.II.2012, leg. R. Müller, 1 ex. TMSA. – South Africa, Eastern Cape, Willowmore, I.1954, leg. F. Zumpt, 7 ex. NHMB, 2 ex. SMNS.

#### Type locality.

“Willowmore”.

#### Distribution.

South Africa (Koch 1953); Tanzania, Zambia, Botswana, Namibia (new records).

**Figures 9–13.**
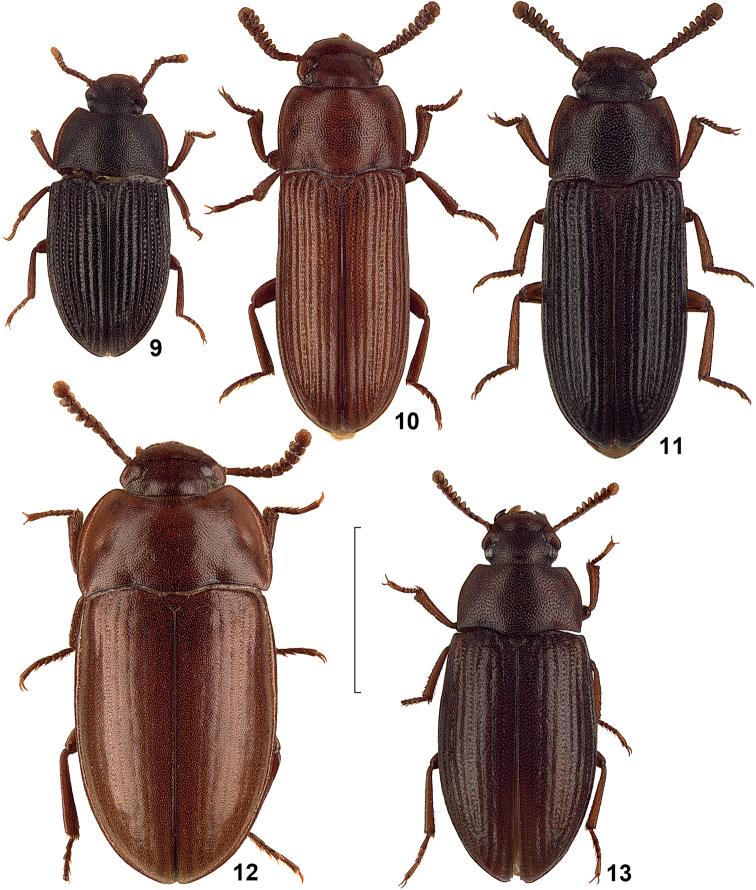
Dorsal view of African species of the genus *Alphitobius*. **9** *Alphitobius kochi*, non-type Togo, SMNS **10** *Alphitobius lucasorum*, paratype, TMSA **11** *Alphitobius parallelipennis*, non-type Angola, SMNS **12** *Alphitobius acutangulus*, non-type Sudan, SMNS **13** *Alphitobius karrooensis*, non-type RSA, TMSA. Scale: 2 mm.

### Alphitobius kochi

Ardoin, 1958

http://species-id.net/wiki/Alphitobius_kochi

Figs 9 , 25

#### Type specimens examined.

Cameroon, N’Kongsamba, XI.1956, leg. J. Cantaloube, 4 paratypes TMSA, 2 paratypes NHMB, 1 paratype MNB, 1 paratype ZSM.

#### New material.

Cameroon, Bambui, 9 miles NE Bamenda, 1450 m, 29.X.1966, leg. E. S. Ross & K. Lorenzen, 1 ex. CRA. – Cameroon, Doala, 10 m, 20.X.1966, leg. E. S. Ross & K. Lorenzen, 7 ex. CRA, 2 ex. SMNS, 1 ex. CRG. – Ivory Coast, 10 miles SE Touba, 420 m, 20.VIII.1966, leg. E. S. Ross & K. Lorenzen, 12 ex. CRA, 2 ex. SMNS. – Liberia, Cari Suakoko, 17.III.1988, leg. F.-T. Krell, 1 ex. SMNS. – Togo/Ghana, border area, Brou fou, 27.X.1984, leg. K. Erber, 1 ex. SMNS.

#### Type locality.

“N’Kongsamba”.

#### Distribution.

Cameroon (type locality), Ivory Coast (Ardoin 1969, Bremer and Girard 1996); Liberia, Togo, Ghana (new records).

### Alphitobius laevigatus

(Fabricius, 1781)

http://species-id.net/wiki/Alphitobius_laevigatus

Figs 14 , 26

Opatrum laevigatus Fabricius, 1781
Tenebrio mauritanicus Fabricius, 1792, syn.
Helops picipes Panzer, 1794, syn.
Helops piceus Olivier, 1795, syn.
Alphitobius granivorus Mulsant & Godart, 1868, syn.
Cataphronetis striatulus Fairmaire, 1869, syn.
Microphyes rufipes MacLeay, 1873, syn.
Alphitobius ruficolor Pic, 1925, syn.

#### New material.

Somalia, Eil (Nogal), III./IV.1938, leg. S. Venzo, 1 ex. TMSA. – Niger, Maradi, 5.–7.VIII.1981, leg. R. Grimm, 1 ex. CRG. – Niger, Niamey, 9. –14.VIII.1989, leg. R. Grimm, 2 ex CRG. – Ghana, Ashanti Region, Kumasi, Nhiasu, 16.V.1967, leg. S. Endrödy-Younga, 1 ex. TMSA. – Cameroon, Longji, 1905, leg. H. Paschen, 1 ex. MNB. – Cameroon, Sasse-Buea, IV.–V.1951, collector unknown, 1 ex. CRA. – Congo, Oubangui-Chari, no further data, leg. Škulina, 1 ex. NMP. – Kenya, no further data, leg. Škulina, 1 ex. NMP. – Tanzania, Daressalam, Pangani, about 1930, leg. R. Regner, 6 ex. MNB. – Tanzania, Pangani to Tanga, VI.1891, leg. Conradt, 2 ex. MNB. – Tanzania, Massa to Tanga, 14.VII.–6.VIII.1891, leg. Conradt, 3 ex. MNB. – Madagascar, Fianarantsoa Prov., Ranamafona, 29.XI.–2.XII.1995, leg. I. Jeniš, 1 ex. CRG. – Madagascar, Ambovombe Distr., Faux Camp, I.1956, leg. C. Koch, 2 ex. TMSA. – Namibia, Fish River Canyon, Ai-Ais, 250 m, 19.XI.1993, leg. M. Uhlig, 1 ex. MNB. – South Africa, Limpopo, Krüger NP, Skukuza, 29.I.1994, leg. S. Endrödy-Younga, 1 ex. TMSA. – South Africa, Limpopo, Krüger NP, Skukuza, 8.–22.XII.2009, leg. J. Harrison, 2 ex. TMSA. – South Africa, Limpopo, Krüger NP, Skukuza, VIII.1992, leg. L. Braack, 10 ex. TMSA. – South Africa, Gauteng, Pretoria, 28.XI.1999, leg. B. Dombrowsky, 1 ex. TMSA. – South Africa, Cape Town, 1965, leg. Dickson, 2 ex. TMSA.

#### Type locality.

“Noua Zelandia”.

#### Distribution.

Cosmopolitan.

**Figures 14–17.**
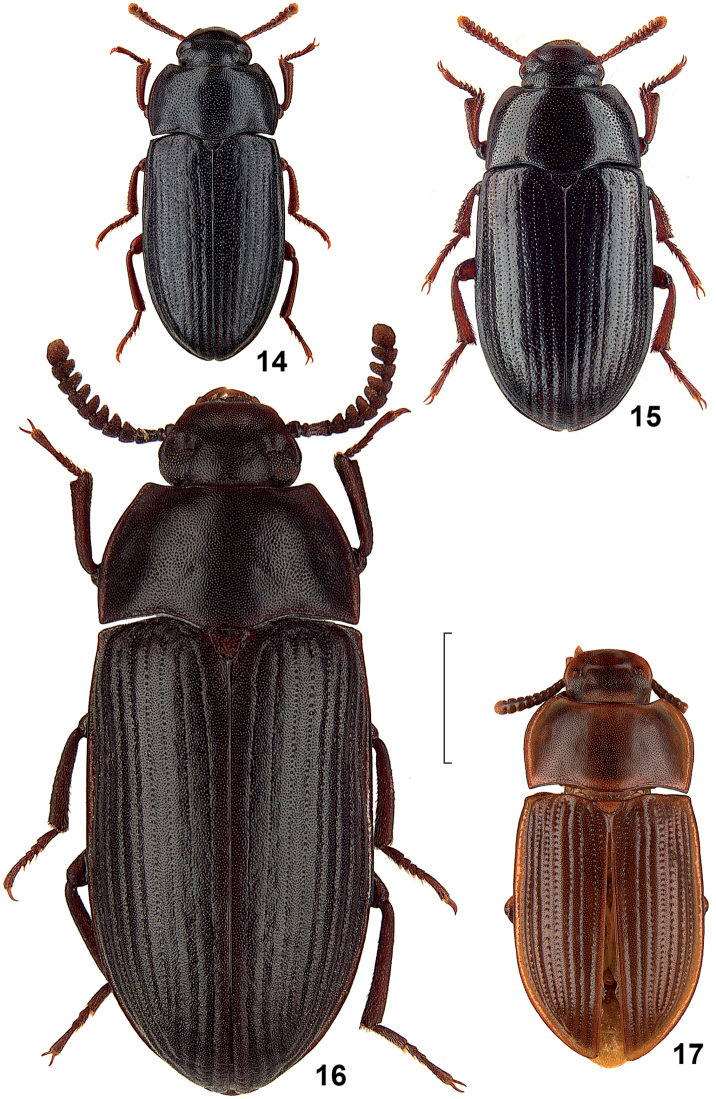
Dorsal view of African species of the genus *Alphitobius*. **14** *Alphitobius laevigatus*, non-type Germany, SMNS **15** *Alphitobius diaperinus*, non-type Germany, SMNS **16** *Alphitobius capitaneus* sp. n., holotype, CRG **17** *Alphitobius limbalis*, doubtful cotype, NHMB. Scale: 2 mm.

**Figures 18–32.**
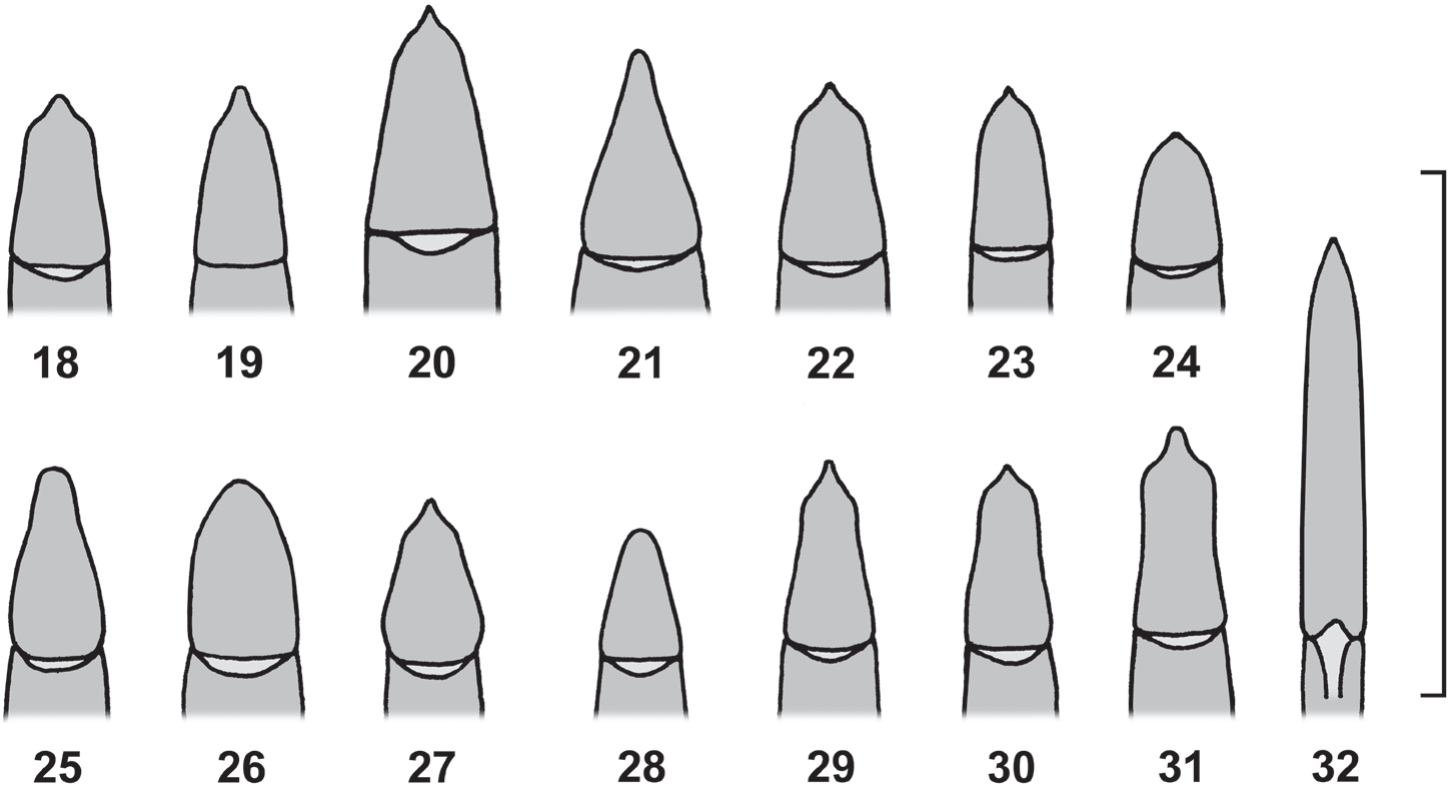
Apicale of aedeagus of African species of the genus *Alphitobius* and *Ulomoides*. **18** *Alphitobius acutangulus*, non-type Sudan, SMNS **19** *Alphitobius arnoldi* nom. n., non-type Tanzania, SMNS **20** *Alphitobius capitaneus* sp. n., holotype, CRG **21** *Alphitobius crenatus*, non-type Madagascar, SMNS **22** *Alphitobius diaperinus*, non-type Zambia, SMNS **23** *Alphitobius hobohmi*, non-type Angola, SMNS **24** *Alphitobius karooensis*, non-type Botswana, SMNS **25** *Alphitobius kochi*, non-type Ivory Coast, SMNS **26** *Alphitobius laevigatus*, non-type Nepal, SMNS **27** *Alphitobius leleupi*, non-type Congo, SMNS **28** *Alphitobius lucasorum*, paratype, SMNS **29** *Alphitobius parallelipennis*, non-type Zambia, CRG **30** *Alphitobius rugosulus*, non-type Tanzania, SMNS **31** *Alphitobius viator*, non-type Zambia, CRG **32** *Ulomoides basilewskyi* comb. n., holotype, MRAC. Scale: 1 mm.

### Alphitobius lamottei

Ardoin, 1963

http://species-id.net/wiki/Alphitobius_lamottei

Fig. 2

#### New material.

Ivory Coast, Bingerville, V.1962, leg. J. Decelle, 1 ex. NHMB. – Guinea, Gbakoré, XII.1983, leg. C. Girard, 2 ex. TMSA (det. Bremer). – Guinea, Mt. Nimba, Keoulenta, 12.I.1984, leg. C. Girard & M. Lamotte, 1 ex. TMSA (det. Bremer). – The Gambia, Kabafita Forest Park, 19.VII.2000, leg. R. Grimm, 1 ex. CRG. – Central African Republic, 35 km E Ndele, 450 m, 18.V.2009, leg. J. Halada, 1 ex. SMNS.

#### Type locality.

“Mt. Nimba”.

#### Distribution.

Guinea (type locality); Senegal (Ardoin 1963a); Ivory Coast (Ardoin 1969, Bremer and Girard 1996); The Gambia (Grimm 2002); Central African Republic (new record).

### Alphitobius leleupi

Koch, 1953

http://species-id.net/wiki/Alphitobius_leleupi

Figs 6 , 27

#### Type specimens examined.

Congo, Massif de Kundelungu, 14.XII.1949, leg. N. Leleup, 29 paratypes TMSA, 2 paratypes SMNS, 1 paratype CRG, 1 paratype ZSM.

#### New material.

Congo, 57 miles N Popokabaka, 3.VIII.1957, leg. E. S. Ross & R. E. Leech, 2 ex. CRA, 1 ex. SMNS.

#### Type locality.

“Kundelungu”.

#### Remarks.

Among the type series in TMSA, the missing holotype of *Alphitobius parallelipennis* was found, see remarks under that species.

#### Distribution.

Congo, Kenya (Koch 1953).

### Alphitobius limbalis

Fairmaire, 1901

http://species-id.net/wiki/Alphitobius_limbalis

Fig. 17

#### Type specimens examined.

Without any data, 1 female “cotype” NHMB (Gebien collection).

#### Remarks.

It seems doubtful to the authors, if the above listed female (body length 6 mm) without any data is really a type specimen. Other material of this taxon is unknown to the authors. The species is said to be similar to *Alphitobius luctuosus* (synonym of *Alphitobius crenatus* (Klug, 1833) (Fairmaire 1901), but is characterised by larger body size (6 mm), rounder pronotum and larger punctures in elytral striae.

#### Type locality.

“Bélumbé”.

#### Distribution.

Madagascar.

### Alphitobius lucasorum

Bremer, 1985

http://species-id.net/wiki/Alphitobius_lucasorum

Figs 10 , 28

#### Type specimens examined.

Sudan, N Darfur Prov., El Geneina, 7.–15.VI.1978, leg. I. Abuzinid, 1 paratype SMNS, 1 paratype TMSA, 1 paratype ZSM. – Ghana, Northern Prov., Nyankpala, 15 km W Tamale, 1.–30.IV.1970, leg. S. Endrödy-Younga, 2 paratypes TMSA.

#### Type locality.

“El Geneina”.

#### Distribution.

Cameroon, Nigeria, Ghana, Senegal, Sudan (type locality) (Bremer and Girard 1996); Chad (Bremer 1985).

### Alphitobius niger

Ferrer, 1983

http://species-id.net/wiki/Alphitobius_niger

#### Type specimens examined.

None, see remarks.

#### Remarks.

Unfortunately, this species was overlooked during the study, and was added here only during the review process. The species is said to be similar to *Alphitobius ulomoides*, for diagnosis and figures see Ferrer (1983). Genal canthus projecting outwards beyond contours of eyes, pronotum widest near base, aedeagus with acute apicale, body length 8 mm.

#### Type locality.

“Lake Manyara”.

#### Distribution.

Tanzania (type locality).

### Alphitobius parallelipennis

Koch, 1953

http://species-id.net/wiki/Alphitobius_parallelipennis

Figs 11 , 29

#### Type specimens examined.

Congo, Lulua, Tshibamba, XII.1931, leg. G. F. Overlaet, holotype MRAC. – Congo, Elisabethville, 7.XI.1923, leg. C. Seydel, 1 paratype TMSA.

#### New material.

Angola, Huila Prov., 75 km N Kaconda, 1640 m, 10.XI.2011, leg. R. Müller, 1 ex. TMSA. – Angola, Huila Prov., 10 km S Kacula, 1560 m, 12.–13.XI.2011, leg. R. Müller & P. Schüle, 1 ex. TMSA, 1 ex. SMNS. – Angola, Huila Prov., 15–20 km S Caluquembe, 6.–7.XI.2011, leg. R. Müller & P. Schüle, 2 ex. TMSA, 2 ex. SMNS. – Angola, Huila Prov., 3.5 km SW Negola, 8.XII.2012, leg. P. Schüle, 2 ex. SMNS. – Zambia, 150 km S Kasemba, 11.XI.2002, leg. F. Wachtel, 11 ex. CRG. – Zambia, 40 km NE Livingstone, Mabula Camp, 14.XI.2002, leg. F. Wachtel, 1 ex. CRG. – Zambia, Lusaka, Kafue River, 1200 m, 22.XI.–2.XII.1987, leg. R. Mourglia, 4 ex. ZSM. – Zambia, Northern Prov., Chipona Falls, 30 km S Chinsali, 5.–6.XII.2002, leg. F. & L. Kantner, 1 ex. SMNS. – Zambia, Copperbelt Prov., NW Kapiri Mposhi, 8.XII.2002, leg. F. & L. Kantner, 1 ex. SMNS. – Zambia, Central Prov., 25 km NE Lilemone, 1250 m, 5.XII.2010, leg. F. Génier, 6 ex. CNC. – Zambia, Lusaka Prov., 9 km E Mulalika, 1100 m, 24.XI.2010, leg. F. Génier, 1 ex. CNC. – South Africa, Limpopo, Krüger NP, Shingwedzi, 19.–20.XI.1961, leg. Vári & Rorke, 1 ex. TMSA. – South Africa, Limpopo (labelled as Transvaal), Manyeleti Game Reserve, 18.XI.1987, leg. T. van Viegen, 1 ex. TMSA.

#### Type locality.

“Lulua, Tshibamba”.

#### Remarks.

Bremer (1985) already assumed, that some specimens of the type series of *Alphitobius leleupi* and *Alphitobius parallelipennis* in TMSA are mislabelled, and that the actual depository of the holotype of *Alphitobius parallelipennis* seems unknown, at least it is not present in MRAC as published in the original description. De Meyer (VII.2013 in an email to the senior author) confirmed, that the holotype is lacking in MRAC with the remark “non renvoyé par Koch”. During the last visit of the senior author in TMSA, the mixture of locality and type labels could be confirmed, and also corrected. The holotype of *Alphitobius parallelipennis* could be recognised without any doubts among the type series of *Alphitobius leleupi*, although mislabelled, and was transferred with correct secondary labels from TMSA to MRAC.

#### Distribution.

Congo (Koch 1953); Angola, Zambia, northeastern South Africa (new records).

### Alphitobius rugosulus

Koch, 1953

http://species-id.net/wiki/Alphitobius_rugosulus

Figs 7 , 30

#### Type specimens examined.

None, not in TMSA.

#### New material.

Ethiopia, Oromia, 6.5 km SE Chichilla, 1550 m, 17.V.2012, leg. F. Wachtel, 1 ex. CRG. – Tanzania, Dodoma Prov., 15 km N Dodoma, 1550 m, 19.XII.2006, leg. F. Kantner, 1 ex. SMNS. – Tanzania, Dodomo Prov., 70 km N Dodoma, 1350 m, 17.XII.2006, leg. F. Kantner, 1 ex. SMNS.

#### Type locality.

“Campi Simba”.

#### Distribution.

Kenya (Koch 1953); Ethiopia, Tanzania (new records).

### Alphitobius viator

Mulsant & Godart, 1868

http://species-id.net/wiki/Alphitobius_viator

Figs 8 , 31

Uloma rufula Fairmaire, 1883, syn.

#### New material.

Ethiopia, Ambo, 3.XI.1990, leg. L. Medvedev, 1 ex. SMNS. – Sudan, North Dafur, El Geneina, 10.VII.1978, leg. I. Abuzinid, 1 ex. ZSM (det. Kaszab). – Ivory Coast, Bingerville, 1962–1964, leg. J. Decelle, 6 ex. MRAC (det. Ardoin). – Ivory Coast, Kafolo/Comoé, 21.IV.1988, leg. F.-T. Krell, 1 ex. SMNS. – Burkina Faso (labelled as Haute Volta), Ouagadongou, no date, leg. Škulina, 1 ex. NMP. – Guinea, N’Zerekoré, 1.–15.XI.1951, leg. S. H. Olsen, 4 ex. NHMB. – Guinea Bissau, Bolama, VI.–XII.1899, leg. L. Fea, 1 ex. NHMB. – Congo, Tshiobo, N’Goy, 3.–4.VII.1926, leg. A. Collart, 2 ex. TMSA, 1 ex. SMNS. – Central African Republic, Uam, Bosum, IV.–VI.1914, leg. Tessmann, 11 ex. MNB. – Central African Republic, 40 km SW Bandoro, 530 m, 14.IV.2010, leg. Halada, 1 ex. SMNS. – Cameroon, Jaunde, X.1914, leg. Tessmann, 1 ex. MNB. – Kenya (labelled as Afr. or.), Ikutha, no further dates, 7 ex. MNB. – Kenya, Mt. Elgon, eastern slope, 2200 m, 23.–27.III.1988, leg. H. J. Bremer, 1 ex. ZSM. – Tanzania, 2 miles SE Mkomazi, 1500 ft. (460 m), 7.I.1970, leg. M. E. Irwin & E. S. Ross, 1 ex. CRA. – Malawi, Salima, 5.–6.I.2002, leg. J. Bezděk, 1 ex. SMNS. – Zambia (labelled as N Rhodesia), Lochinvar, near Monze, 13.–26.X.1962, leg. F. Zumpt, 3 ex. NHMB. – Zambia, Northern Prov., Chipona Falls, 30 km S Chinsali, 5.–6.XII.2002, leg. F. Kantner, 1 ex. SMNS. – Zambia, 150 km S Kasempa, 11.XI.2002, leg. F. Wachtel, 1 ex. CRG. – Zimbabwe, 60 km N Bulawayo, Maraposa Road, 3.XII.1998, leg. M. Snižek, 1 ex. ZSM. – Botswana, 10 km N Martin’s Drift, 7.I.2010, leg. P. Schüle, 1 ex. SMNS. – Angola, Sá da Bandeira, 2.X.1949, leg. B. Malkin, 1 ex. CRA. – Angola, Huambo Prov., 75 km N Caconda near Cuima, 10.–11.XI.2011, leg. P. Schüle, 1 ex. SMNS. – South Africa, Limpopo, Manyeleti Game Reserve, 17.–19.XI.1987, leg. T. van Viegen, 2 ex. TMSA. – South Africa, Limpopo, Naboomspruit, Torino Ranche, 15.I.1990, leg. S. Endrödy-Younga, 1 ex. TMSA. – South Africa, Limpopo, Kruger NP, Skukuza Research Camp, 1.–16.XII.2010, leg. J. Harrison, 1 ex. TMSA. – South Africa, Limpopo, Amatola, Scott Farm, 26.I.1998, leg. R. Müller, 1 ex. TMSA. – South Africa, Limpopo, Thabazimbi, 16.XI.2002, leg. F. Wachtel, 4 ex. CRG. – South Africa, Limpopo, N Makopane (Potgietersrus), Thabaphaspha Farm, 1400 m, 13.–14.XII.2003, leg. R. Müller & W. Schawaller, 1 ex. SMNS, 1 ex. TMSA. – South Africa, Limpopo, Mapungubwe, Little Muck NR, 500 m, 6.–9.XII.2008, leg. W. Schawaller, 1 ex. SMNS. – South Africa, Limpopo, 10 km N Modimolle (Nylstroom), Kuthaba Bush Lodge, 1300 m, 10.–13.XII.2008, leg. W. Schawaller, 2 ex. SMNS. – South Africa, Limpopo, Waterberg, Geelhoutbosch Farm, 15.XII.1997, leg. C. L. Bellamy, 1 ex. TMSA. – South Africa, Limpopo, 15 km NE Klaserie, Guernsey Farm, 18.–30.XII.1985, leg. S. & J. Peck, 1 ex. TMSA. – South Africa, Mpumalanga, Booysendal Farm, 25.X.2000, leg. TMSA staff, 1 ex. TMSA. – South Africa, Gauteng, Tswaing, 17.II.2003, leg. TMSA staff, 1 ex. TMSA. – South Africa, Free State, Bothaville, 15.II.1898, leg. H. Brauns, 5 ex. TMSA. – South Africa, KwaZulu-Natal, SW Magudu, 4.–5.I.2009, leg. P. Schüle, 3 ex. SMNS.

#### Type locality.

“Marseille, importée” (*viator*), “Abyssinie” (*rufula*).

#### Remarks.

We could not clear, if *rufula* Fairmaire, 1883 is a synonym of *viator* Mulsant & Godart, 1868 (as listed in all catalogues), or of *hobohmi* Koch, 1953 (with *rufus* Ardoin, 1976, syn. n.). In some localities (for example Modimolle and Magudu) *Alphitobius viator* was collected together with *Alphitobius hobohmi*.

#### Distribution.

Tropical and southern Africa, the holotype was imported to Marseille in southern France (Mulsant and Godart 1868).

### Key to the species of African *Alphitobius*

Unfortunately, the taxonomic status of *Alphitobius grandis* Fairmaire, 1897 and *Alphitobius limbalis* Fairmaire, 1901 from Madagascar could not be enlightened by the authors, thus both taxa are not included herein. Also not included is *Alphitobius niger* Ferrer, 1983, which was overlooked during the study and included herein only during the review process. Compare also Figs 1–17.

**Table:** 

1	Genal canthus not distinctly projecting outwards beyond contours of eyes	2
–	Genal canthus projecting outwards beyond contours of eyes	5
2	Body length 8.8 mm, pronotum widest in posterior third	*Alphitobius capitaneus* sp. n.
–	Body length ≤ 7.0 mm, pronotum widest near posterior angles	3
3	Lateral margins of pronotum distinctly rounded towards anterior angles (Fig. 2)	*Alphitobius lamottei*
–	Lateral margins of pronotum nearly straight or slightly sinuate narrowing towards anterior angles, thus shape of pronotum conical (Figs 4, 5)	4
4	Lateral margins of pronotum nearly straight, pronotal disc with punctures of similar size	*Alphitobius arnoldi* nom. n.
–	Lateral margins of pronotum slightly sinuate, pronotal disc with large and small punctures of different size	*Alphitobius hobohmi*
5	Lateral margins of pronotum rounded towards posterior angles, pronotum widest in the middle or shortly behind the middle	6
–	Lateral margins of pronotum subparallel, pronotum widest near base	10
6	Elytra without distinct punctural rows, only laterally with traces of punctural rows, elytra with fine microsetation	*Alphitobius acutangulus*
–	Eytra completely with distinct punctural rows, elytra bare or with fine microsetation	7
7	All elytral intervals distinctly convex, nearly keel-like (Figs 7, 9), elytra with fine microsetation	8
–	All elytral intervals flat or only external intervals slightly convex, but not keel-like, elytra bare	9
8	Body length 3–4 mm, apicale of aedeagus with rounded tip (Fig. 25)	*Alphitobius kochi*
–	Body length above 5 mm, apicale of aedeagus with triangular acute tip (Fig. 3)	*Alphitobius rugosulus*
9	Internal punctural rows of elytra not impressed, last 5 antennomeres forming a separated club	*Alphitobius laevigatus*
–	All punctural rows of elytra impressed, last 5 antennomeres not separated from the remaining basal ones	*Alphitobius crenatus*
10	Elytra long and narrow, parallel-sided (Figs 10, 11)	11
–	Elytra shorter and broader, ovate (Figs 6, 8, 13, 15)	12
11	Lateral margins of pronotum regularly rounded, anterior corners not prominent, apicale of adeagus with rounded tip (Fig. 28)	*Alphitobius lucasorum*
–	Lateral margins of pronotum parallel in basal part, anterior corners prominent, apicale of aedeagus triangular with acute tip (Fig. 29)	*Alphitobius parallelipennis*
12	Lateral margins of pronotum straight and parallel in basal part, pronotum widest near base	13
–	Lateral margins of pronotum rounded towards posterior angles, pronotum widest in middle	14
13	Dorsal side blackish and shining, base of pronotum unbordered in the middle, apicale of aedeagus shorter (Fig. 22)	*Alphitobius diaperinus*
–	Dorsal side brownish and dull, base of pronotum completely bordered, apicale of aedeagus longer (Fig. 31)	*Alphitobius viator*
14	Pronotum convex with rough and confluent punctuation, without slight transverse impression (Fig. 13)	*Alphitobius karooensis*
–	Pronotum more flat and with finer separate punctuation, with a feeble transverse impression (Fig. 6)	*Alphitobius leleupi*

### New combinations

#### Ulomoides basilewskyi

(Ardoin, 1969) comb. n.

http://species-id.net/wiki/Ulomoides_basilewskyi

Figs 3 , 32

Alphitobius basilewskyi Ardoin, 1969

##### Type specimens examined.

Ivory Coast, Bingerville, VI.1962, leg. J. Decelle, male holotype MRAC. – Same locality and collector as holotype, but IV.1962–III.1963, 3 paratypes MRAC.

##### Type locality.

“Bingerville”.

##### Remarks.

*Ulomoides basilewskyi* (Ardoin, 1969) possesses distinctly crenulated outer margin of all tibiae (see Ardoin 1969), which is characteristic for some species of *Ulomoides* Blackburn, 1888 (Hinton 1947, under *Martianus* Fairmaire, 1893), but not for *Alphitobius*. In the structure of tibiae, the shape of body, eyes, and antennae *Ulomoides basilewskyi* resembles *Ulomoides dermestoides* (Chevrolat, 1878). The striking long apicale of the aedeagus (Fig. 32) resembles those of some *Ulomoides*, but differs distinctly from those of the *Alphitobius* species (Figs 18–31).

##### Distribution.

Ivory Coast.

#### Peltoides (Micropeltoides) opacus

(Gerstaecker, 1871) comb. n.

http://species-id.net/wiki/Peltoides_opacus

Alphitobius opacus Gerstaecker, 1871
Diaclina opaca (Gerstaecker, 1871) sensu Gebien (1940)
Peltoides (Micropeltoides) crypticoides Pic, 1916, syn. n.

##### Type specimens examined.

Ugano, leg. v. d. Decken, no. 56752, no further data, holotype of *opacus* MNB. – Fort Crampel, no further data, holotype of *crypticoides* MNHN.

##### New material.

Benin, Kokora, 52 km N Save, 21.VI.2001, leg. F. & L. Kantner, 1 ex. SMNS. – Uganda, Bwamba Forest, 2500 ft. (760 m), III.1948, leg. J. G. Williams, 1 ex. SMNS. – Angola, Huila Province, 20 km S Caluquembe, 6.–7.XI.2011, leg. P. Schüle, 1 ex. SMNS. – Guinea (labelled as French Guinea), Region Kindia, Ségueia, 10.V.1951, leg. J. Bechyné, 4 ex. NHMB, 1 ex. CRG. – SE Cameroon, Lolodorf, leg. L. Conradt, 1895, 1 ex. NHMB. – Tanzania (labelled as Deutsch Ostafrika), [residual label unreadable], 1 ex. NHMB. – Zambia, Ikengele, Nchila Reserve, 6.XI.2002, leg. F. Wachtel, 1 ex. CRG.

##### Type localities.

“Ugano-Berge” (*opacus*), “Fort Crampel (Kaga Bandora)” (*crypticoides*).

##### Remarks.

The examination of the type of *Alphitobius opacus* Gerstaecker, 1871 shows, that the original assignment to *Alphitobius* is wrong and that this species must be transferred to the genus *Peltoides* Laporte, 1832, subgenus *Micropeltoides* Pic, 1916, because of entirely different body shape, different shape of antennomeres, and different shape of male genitalia with the base of basale not asymmetrical as in Alphitobiini. The type of *Peltoides (Micropeltoides) crypticoides* Pic, 1916 fully coincide with *opacus*, and is thus a junior synonym.

##### Distribution.

Tanzania (type locality *opacus*), Central African Republic (type locality of *crypticoides*); Senegal (Ardoin 1963b), Ivory Coast (Ardoin 1969), Mali, The Gambia (Grimm 2002, all under *Peltoides (Micropeltoides) crypticoides*); Benin, Cameroon, Guinea, Uganda, Angola, Zambia (new records).

## Supplementary Material

XML Treatment for Alphitobius acutangulus

XML Treatment for Alphitobius arnoldi

XML Treatment for Alphitobius capitaneus

XML Treatment for Alphitobius crenatus

XML Treatment for Alphitobius diaperinus

XML Treatment for Alphitobius grandis

XML Treatment for Alphitobius hobohmi

XML Treatment for Alphitobius karrooensis

XML Treatment for Alphitobius kochi

XML Treatment for Alphitobius laevigatus

XML Treatment for Alphitobius lamottei

XML Treatment for Alphitobius leleupi

XML Treatment for Alphitobius limbalis

XML Treatment for Alphitobius lucasorum

XML Treatment for Alphitobius niger

XML Treatment for Alphitobius parallelipennis

XML Treatment for Alphitobius rugosulus

XML Treatment for Alphitobius viator

XML Treatment for Ulomoides basilewskyi

XML Treatment for Peltoides (Micropeltoides) opacus
